# Fluorotic Enamel Susceptibility to Dental Erosion and Fluoride Treatment

**DOI:** 10.1590/0103-6440202305595

**Published:** 2023-12-22

**Authors:** Cristiane Araújo Maia Silva, Frederico Barbosa de Sousa, Esperanza Angeles Martinez-Mier, Basílio Rodrigues Vieira, Johnatan Meireles do Nascimento, Anderson Takeo Hara

**Affiliations:** 1 Federal University of Paraiba, Joao Pessoa, Paraíba, Brazil.; 2 Department of Morphology, Health Sciences Center, Federal University of Paraiba, Joao Pessoa, Paraíba, Brazil; 3 Department of Cariology, Operative Dentistry and Dental Public Health, Indiana University School of Dentistry, Indianapolis, IN, USA

**Keywords:** Dental Fluorosis, Dental Erosion, Fluoride, Dental Enamel, Tooth Wear

## Abstract

The purpose of this *in vitro* study was to test the hypothesis that fluoride treatment can prevent dental erosion on fluorotic enamel of different severities. It followed a 3×2 factorial design, considering a) fluorosis severity: sound (TF0, Thylstrup-Fejerskov Index), mild (TF1-2), moderate (TF3-4); and b) fluoride treatment: 0 (negative control) and 1150ppmF. Human molars with the three fluorosis severities (n=16, each) were selected and randomly assigned to the two fluoride treatments (n=8). Enamel blocks (4×4mm) were prepared from each tooth and subjected to a dental erosion cycling model, for 10 days. The daily cycling protocol consisted of erosive challenges (1% citric acid, pH 2.4), interspersed by periods of immersion in artificial saliva, and three 2-minute treatments with either 0 or 1150ppm F. The enamel volume loss (mm^3^) was calculated by subtracting values obtained by microtomography before and after cycling. Two-Way ANOVA showed no significant interaction between fluorosis severity and fluoride treatment (p=0.691), and no significant effect for either fluorosis severity (TF0 mean±standard-deviation: 13.5(10^-2^±0.42(10^-2^, TF1-2: 1.50(10^-2^±0.52(10^-2^, TF3-4: 1.24(10^-2^±0.52(10^-2^, p=0.416) or treatment (0ppmF: 1.49(10^-2^±0.53(10^-2^; 1150ppmF: 1.21(10^-2^±0.42(10^-2^; p=0.093), when evaluated independently. Considering the limitations of this *in vitro* study, the presence and severity of fluorosis in enamel do not appear to affect its susceptibility to dental erosion. Fluoride treatment was not effective in preventing the development of dental erosion in both sound and fluorotic enamel substrates under our experimental conditions.

## Resumo

Este estudo in vitro foi testou a hipótese de que o tratamento com flúor pode prevenir a erosão dentária no esmalte fluorótico de diferentes severidades. O objetivo deste estudo foi: investigar o efeito protetor dos fluoretos contra a erosão e abrasão simuladas no esmalte fluorótico. Seguiu um desenho fatorial 3×2, considerando a) severidade da fluorose em 3 níveis: hígido (TF0, Índice Thylstrup-Fejerskov), suave (TF1-2), moderada (TF3-4); b) tratamento com flúor: 0 (controle negativo) e 1150ppmF. Molares humanos com as três severidades de fluorose (n=16, cada) foram selecionados e distribuídos aleatoriamente para os dois tratamentos com flúor (n=8). Blocos de esmalte (4×4mm) foram preparados a partir de cada dente e submetidos a um modelo de ciclo de erosão dentária, por 10 dias. O protocolo de ciclagem diária consistiu em seis desafios erosivos de 5 minutos (1% de ácido cítrico, pH 2,4), intercalados por seis períodos de imersão em saliva artificial e três tratamentos de 2 minutos com 0 ou 1150ppmF. O volume do esmalte perdido foi calculado subtraindo o perfil superficial 3D obtido por microtomografia antes e depois da ciclagem. A ANOVA de dois fatores não mostrou interação significativa entre a severidade da fluorose e o tratamento com flúor (p = 0,691) e nenhum efeito significativo para a severidade da fluorose (TF0 média+/desvio padrão: 13,5(10^-2^±0,42(10^-2^, TF1-2: 1,50(10^-2^±0,52(10^-2^, TF3-4: 1,24(10^-2^±0,52(10^-2^, p=0,416) ou tratamento (0: 1,49(10^-2^±0,53(10^-2^; 1150ppmF: 1,21(10^-2^±0,42(10^-2^, p=0,093), quando avaliados independentemente. Considerando as limitações deste estudo in vitro, a presença e severidade da fluorose no esmalte não parece afetar sua suscetibilidade à erosão dentária. O tratamento com flúor não foi eficaz na prevenção do desenvolvimento da erosão dentária em esmalte hígido e fluorótico, sob as condições experimentais utilizadas.

## Introduction

Excessive exposure to fluoride during the enamel formation period can lead to enamel fluorosis ^1^. Studies have shown that enamel with fluorosis is hypomineralized and exhibits higher fluoride content ^2^,^3^. Fluorotic enamel is more porous, which lowers its mechanical resistance; but it is also rich in fluoride, which could potentially increase its resistance to demineralization ^4^. Fluoride compounds can reduce tooth dissolution and, in some cases, increase tooth resistance to erosive acids ^5^. Although some *in vitro* studies have shown that higher fluoride content in enamel can protect it from demineralization due to caries simulation, no studies have tested if fluorotic enamel presents a different susceptibility against erosive tooth wear (ETW) ^6^.

The control of ETW should focus on preventive measures. Thus, besides avoiding tooth exposure to acids, the use of fluoride products has been recommended ^7^. Fluoride acts on the erosive process differently from what has been reported for caries ^7^ with the impact of fluoride in the dental erosion context being much less prominent. It has been suggested that higher concentration and frequency of application of fluoride treatment may increase protection against dental erosion, although this effect is limited ^8^. Sodium fluoride has shown some efficacy against dental erosion, which is most likely achieved by physically protecting tooth surfaces with calcium fluoride (CaF_2_) deposits ^8^ ^,^ ^9^. This layer acts as a physical barrier to acids and as a reservoir of calcium and fluoride ions that are released at low pH, increasing the saturation of the enamel apatite. Data from *in vitro* and situ studies showed that topical application of fluoride can protect dental substrates against erosive attacks ^10^ ^,^ ^11^. Specifically for toothpastes, NaF has shown some protection, with moderate evidence ^12^ especially when 1450 ppm fluoride was used in primary teeth ^13^ ^,^ ^14^. However, no information is available on its protective effect on fluorotic enamel. This study tested the following hypotheses: 1) whether fluorotic enamel of different severities presents different susceptibility to dental erosion *in vitro*; and 2) whether treatment with a fluoride solution can prevent dental erosion progression on fluorotic enamel.

## Materials and methods

### Experimental Design

**Figure 2:**
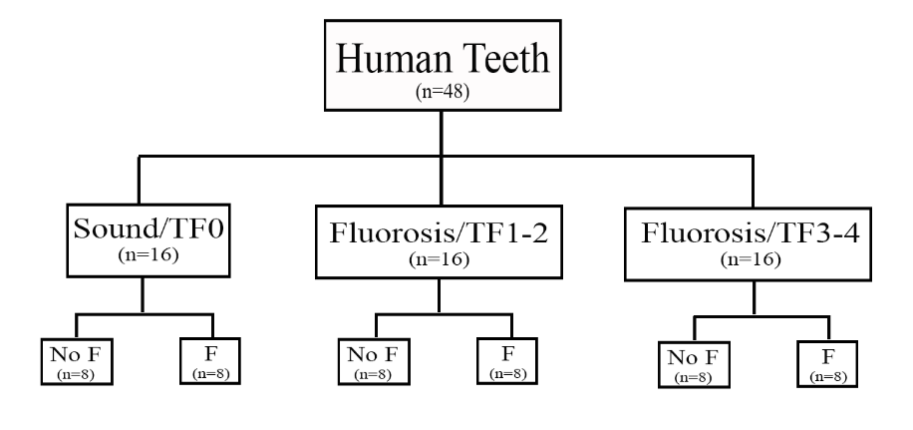
Experimental design of the study (TF: Fluorosis Index, n: sample size, F: treatment with fluoridated solution, No F: treatment with a no-fluoridated solution.

This was a quantitative experimental analytical study with a direct observation technique ^15^. It followed a 3×2 factorial design using an *in vitro* dental erosion simulation model. Experimental factors were a) fluorosis severity at 3 levels: sound (TF0, Thylstrup-Fejerskov Index), mild (TF1-2), moderate (TF3-4); b) fluoride treatment at 2 levels: 0 (negative control) and 1150 ppm F (from NaF). The dental erosion simulation was performed for 10 days. Forty-eight human teeth with the three levels of fluorosis severity (n=16) were selected, prepared, and randomly assigned to each of the fluoride treatments (n=8), generating six groups (Figure 2). The sample size was based on Marin et al. (2016), who studied the susceptibility of fluorotic human enamel to an in vitro cariogenic challenge and reported a Hedge G effect magnitude of 1.375, between the most contrasting groups in terms of expected differences (TF 0 and TF 3-4). Using a power of 80%, a significance level of 5%, and one-tailed directionality, a sample size of 8 samples per group was calculated. The response variable enamel surface loss (in micrometers) was assessed by micro-computed tomography (micro-CT).

### Teeth Selection and Specimen Preparation

A total of 48 unidentified extracted human permanent molars were collected, including 16 sound (control) teeth and 32 teeth with the studied fluorosis severity levels. Enamel fluorosis was visually assessed by TFI, by a previously trained and calibrated examiner (CAMS). They were examined using a stereomicroscope at 2× magnification. Those with the presence of visual caries lesions, restorations, enamel fissures/cracks, and enamel fractures were excluded. The teeth were carefully cleaned using a periodontal curette to remove soft tissue and stored in 0.1% thymol solution, at 4 degrees Celsius until use. Enamel blocks (4×4×2 mm) were cut using a microtome (Isomet Low-Speed Saw, Buehler). The enamel surface was covered with adhesive tape, except for a circular area (1 mm in diameter) in the center, which was exposed to the experimental treatments. After preparing the specimens, they were assigned to either fluoride treatment or water using balanced randomization, within each of the TFI scores.

### Dental Erosion Cycling

**Figure 1:**
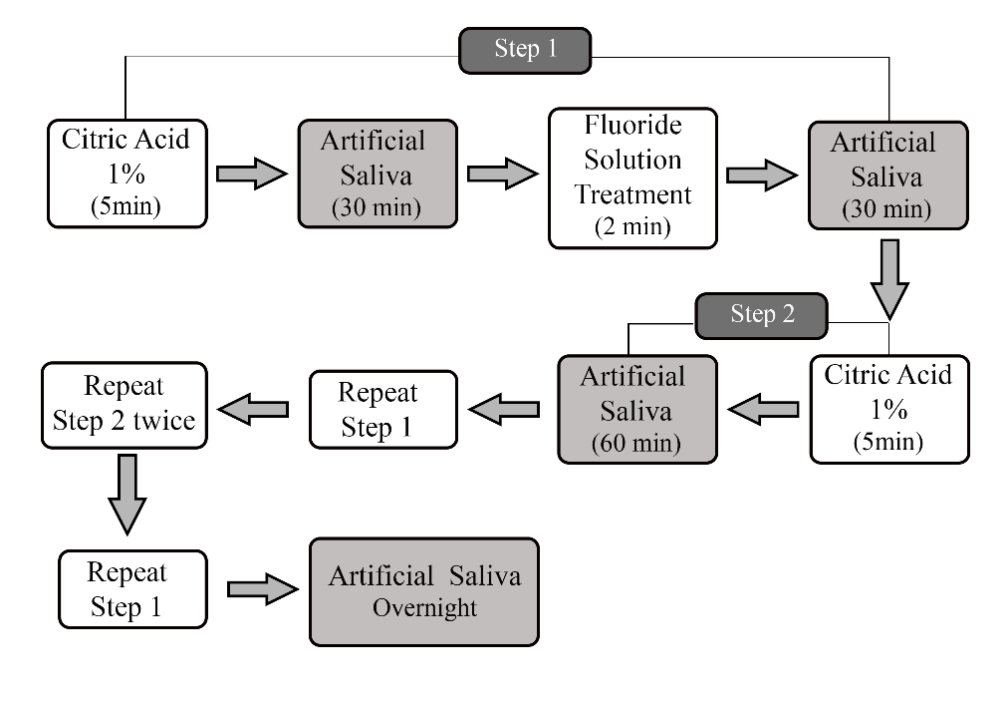
Daily sequence of erosion cycling with citric acid and treatment with 1150ppm F solution. Step One: Citric Acid 1% (5 min) - Artificial Saliva (30 min) - Fluoride Solution Treatment (2 min) - Artificial Saliva (30 min); Step Two: Citric Acid 1 % (5 min) - Artificial Saliva (60 min), repeat Step One, repeat Step Two twice, repeat Step One, Artificial Saliva overnight.

The specimens were submitted to a dental erosion cycling model (Scaramucci et al., 2013). The daily cycle consisted of two steps. In step 1, specimens were immersed in 1% citric acid solution (pH ~2.4, 5 min, 5ml/sample, without agitation), immersed in artificial saliva (30 min, replenished daily, under gentle agitation, room temperature), treated with either 0 or 1150 ppm fluoride solution (2 min) and immersed in artificial saliva (30 min). In step 2, the samples were immersed in 1% citric acid solution (pH ~ 2.4, 5 min, 5ml/ sample, without agitation), and immersed in artificial saliva (60 min). After demineralization and remineralization, the specimens were washed with distilled water and carefully dried with a paper towel. The steps were repeated following the cycling of the reference study, three times each step throughout the day. The specimens were rinsed with deionized water for 10 seconds and stored in artificial saliva with agitation at 150 rpm overnight. These daily procedures were repeated for 10 days (Figure 1).

### Microtomography Analysis (Micro CT)

Samples were scanned on micro-CT and three-dimensional images were obtained using a Skyscan 1172 equipment (Bruker, United States of America). Micro CT was used to quantify the enamel volume loss. Samples were analyzed before and after the cycling phase. Images were acquired using the following parameters: 4.88-μm pixel, 100-kV voltage (a peak emission energy of 59 keV; according to the manufacturer), 100-μA amperage, 180°-rotation, 3-frame average, 0.5°-rotation step, and AlCu filter. The images were reconstructed and converted to bitmap by nRecon software v1.5.23 with 10-smoothing, 18-ring artifact reduction, and 25% beam-hardening. The images (pre- and post-cycling) were aligned with the DataViewer morphometric visualization software (Bruker MicroCT), and the enamel loss was quantified using the co-registration technique (MeshLab and Rhinoceros). Pre and post-erosive challenge images were positioned in an overlapping manner and the co-registration was performed using the MeshLab software (ISTI - CNR, Italy). Using Rhinoceros 3D software (Robert McNeel & Associates, United States of America), the three-dimensional co-registered images were positioned side by side, and cylinders measuring 0.4 mm in radius and 1.5 mm deep were inserted, one in the area submitted to the challenge, and three in control areas, so that the cylinders were positioned at the same location in both images. The volume loss calculation was performed by subtracting the baseline enamel volume from the enamel volume measured after treatment.

### Statistical Analysis

Statistical analyses were performed using the software Rstudio (R version 4.2.1). The normality of data was checked by calculating skewness and kurtosis for each group, and those groups presenting skewness and kurtosis within the range of ± 2 from the optimal value (-2 to 2 in skewness; 1-5 in kurtosis) were considered as normally distributed ^16^. Two-way ANOVA was used to study the effects of enamel fluorosis severity (sound, TF1-2, and TF3-4) and treatment (water and fluoride solution), as well as their interaction on enamel volume loss. A significance level of 5% was adopted.

## Results

**Figure 3:**
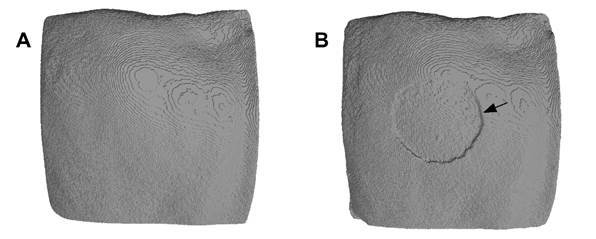
Typical aspects of a specimen at baseline (A) and after (B) the erosion cycling. The eroded area is indicated by an arrow.

Typical aspects under micro-CT of a specimen at baseline and after the erosive challenge are shown in Figure 3. Means (standard deviations) and medians of enamel volume loss (mm3) for each group are presented in Table 1. Samples were lost in 4 groups (with final sample size ranging from 6 to 8 per group) due to failure in the adhesion of the protective tape to the reference/control enamel surface, exposing it to acid. The interaction between the studied factors was not significant (p=0.691). There were no significant differences in enamel volume loss when comparing the different enamel fluorosis severities (p = 0.416), or type of treatment solution (p=0.093).

**Table 1:** Means (± standard deviations) of enamel volume at baseline and after erosion, and of enamel volume loss (mm^3^).

Group	n	Baseline	Post-cycling	Volume Loss
Sound /Control	6	49.63(10^-2^ (±10.87(10^-2^)	48.24(10^-2^ (±10.70(10^-2^)	1.39(10^-2^ (±0.36(10^-2^)
Sound / NaF	6	49.24(10^-2^ (±7.36(10^-2^)	47.92(10^-2^ (±7.13(10^-2^)	1.32(10^-2^ (±0.51(10^-2^)
TFI 1-2 / Control	8	47.47(10^-2^ (±9.93(10^-2^)	45.85(10^-2^ (±10.14(10^-2^)	1.62(10 ^-2^ (±0.63(10^-2^)
TFI 1-2 / NaF	6	50.37(10^-2^ (±2.84(10^-2^)	49.10(10^-2^ (±2.74(10^-2^)	1.33(10^-2^ (±0.31(10^-2^)
TFI 3-4 / Control	8	48.85(10^-2^ (±1.79(10^-2^)	47.42(10^-2^ (±2.04(10^-2^)	1.43(10^-2^ (±0.55(10^-2^)
TFI 3-4 / NaF	7	49.20(10^-2^ (±6.46(10^-2^)	48.17(10^-2^ (±6.46(10^-2^)	1.03(10^-2^ (±0.41(10^-2^)

## Discussion

The study hypothesis that fluoride treatment could interfere with the development of dental erosive-abrasive lesions on fluorotic enamel was rejected. Treatment with fluoride solution did not reduce enamel structure loss, regardless of the severity level of enamel fluorosis. Fluoride did not reduce the enamel loss even on sound enamel. Despite the reduced sample size, the differences between affected groups were small (ranging between 1.00(10^-2^ and 1.60(10^-2^ ± 0.5(10^-2^ mm^3^), therefore not affecting the results of the statistical inferential analyses. Our result contrasts with what has been observed in previous studies ^17^ ^,^ ^18^, and could be possibly explained based on: (i) the higher content of fluoride on the natural (unpolished) enamel surface of sound enamel, and (ii) the limitations of the methods used in this study, involving type and evaluation of the specimen.

In this study, we tested the natural enamel surface of human permanent molars, which could potentially include samples with past episodes of subclinical de- and remineralization at the interface between plaque fluid and the enamel surface. Such episodes are likely to increase the fluoride content in the enamel surface. Therefore, the relatively lower degree of saturation of the mature enamel surface could have increased its resistance to dental erosion, possibly reducing or eliminating the potential protection provided by the fluoridated solution (such as the one used ^19^ against dental erosion in the present study. The treatment with NaF solution can result in the formation of a CaF_2_ layer on the enamel surface; however, even under favorable *in vitro* conditions, only < 40% of the enamel surface showed to be covered by CaF_2_-like particles ^20^ ^,^ ^21^. To date, the question of the amount of time for this CaF_2_ precipitate to form *in vivo* in sound enamel has not been resolved ^22^.

It is possible that different results could be found for different fluoride compounds, such as monovalent and polyvalent fluorides ^17^ therefore, this may be the subject of follow-up studies using similar enamel substrates and dental erosion models. The choice of NaF in our study was because this compound is widely used in oral hygiene products, and it has been shown to reduce (19%) enamel erosive loss when present in mouth rinses ^23^. Since it is assumed that the precipitation of mineral salts depends on the concentrations of the active ingredients in the solution, as well as on the pH, and that applications of fluoride-containing polyvalent metal ions are more effective in preventing dental erosion, it can be suggested that the CaF_2_-like precipitates resulting from the topical application of NaF were readily soluble under mildly erosive conditions *in vitro*. However, they are retained much longer under intraoral acid impact ^8^ where salivary factors may play an important role and can modulate the effectiveness of the fluoride solution clinically ^24^

The vast majority of laboratory studies in the area of dental erosion use flattened and polished dental surfaces, to minimize the biological variation between specimens (mostly on the enamel surface layer) and maximize the ability of evaluation methods to identify differences between experimental factors ^18^ ^,^ ^25^. Our objective was to study the natural fluorotic surfaces, therefore it was imperative to maintain the natural enamel surface. It is reasonable to consider that significant differences in the fluoride treatment effect could have been found if different methods for the assessment of mineral loss were utilized. The study outcome was calculated by the subtraction of the enamel volume assessed before and after the dental erosion simulation, quantifying the enamel structure loss. Methods able to better quantify the mineral content on the remaining enamel (not etched away by the acid challenge), such as microradiography and microhardness, could perhaps indicate higher contents on enamel treated by the fluoride solutions. This remains to be investigated in further studies.

When interpreting the relevance of the findings of this study, it is important to consider the myriad of clinical factors that can directly or indirectly affect tooth wear. Modulating factors such as behavior (brushing frequency, pressure, length, type of toothpaste, type of brush), chemical (fluoride, detergents, acids) and biological (dental substrate, saliva, and dental biofilm), as well as their interaction should be considered ^26^. Therefore, there is a need for further studies controlling for these factors in an isolated and, eventually, interactive way. Future studies in this area should also focus on improving experimental simulations and evaluating dental erosion on natural enamel surfaces. Furthermore, using a larger sample size could increase the robustness of similar types of studies. The resolution (eg.: pixel size of 4.88 μm used) of the evaluation method in detecting enamel surface loss might be considered a limitation of this study, and further studies using techniques with higher resolution would shed some light on this topic.

Considering the limitations of this *in vitro* study, the susceptibility of fluorotic enamel to dental erosion was neither modified by its severity nor by fluoride treatment. Further investigations are needed, focusing on improving experimental conditions and evaluation methods that can minimize observed experimental errors.
